# Discovery of Iron-Chelating Peptides from *Lupinus mutabilis* via Integrated Purification and *In Silico* Validation

**DOI:** 10.3390/foods15081318

**Published:** 2026-04-10

**Authors:** Zayra Vila-Santillán, David Campos, Ana Aguilar-Galvez, Sebastien Carpentier, Thomás Valente de Oliveira, Romina Pedreschi, Rosana Chirinos

**Affiliations:** 1 Instituto de Biotecnología, Research Group on Functional Foods and Nutraceuticals, Universidad Nacional Agraria La Molina, Av. La Molina, La Molina, Lima 12056, Peru; 20221586@lamolina.edu.pe (Z.V.-S.); aaguilar@lamolina.edu.pe (A.A.-G.); 2 Bioversity International, Biodiversity for Food and Agriculture, 3000 Leuven, Belgium; sebastien.carpentier@kuleuven.be; 3 Facility for Systems Biology Based Mass Spectrometry SYBIOMA, KU Leuven, 3001 Leuven, Belgium; 4 Faculdade de Engenharia Química, Universidade de Uberlândia, Patos de Minas 38701-270, MG, Brazil; thomas.oliveira@ufu.br; 5 Escuela de Agronomía, Pontificia Universidad Católica de Valparaíso (PUCV), Calle San Francisco s/n, La Palma, Quillota 2260000, Chile; romina.pedreschi@pucv.cl; 6 Millennium Institute Center for Genome Regulation (CRG), Santiago 8320000, Chile

**Keywords:** iron chelating capacity, protein hydrolyzates, peptides, molecular docking, LC-MS/MS *de novo* sequencing

## Abstract

This study evaluated the iron-chelating capacity (ICC) of *Lupinus mutabilis* protein hydrolysate (LMPH) and its peptide fractions obtained through ultrafiltration and purification by immobilized metal ion affinity chromatography (IMAC) and gel filtration chromatography (GFC). Peptides were identified by LC-MS/MS, and their interactions with Fe^2+^ were analysed using molecular docking. LMPH was produced by enzymatic hydrolysis with Alcalase and subsequently subjected to ultrafiltration to concentrate peptides smaller than 2 kDa. This fraction exhibited higher ICC (35.1 mg Fe^2+^·g^−1^) than the hydrolysate (22.75 mg Fe^2+^·g^−1^). Sequential purification by IMAC and GFC yielded peptide fractions with enhanced ICC values (45.20 and 13.51 mg Fe^2+^·g^−1^). A total of 176 peptides were identified by *de novo* LC-MS/MS sequencing, from which nine were selected based on favourable structure–ICC relationships and absence of predicted toxicity. Molecular docking analysis suggested spatial proximity between Fe^2+^ and the selected peptides. Although stable multi-site binding was not predicted under the applied computational model, the results support the potential of these sequences to interact with Fe^2+^. These findings provide molecular and chemical insights supporting the iron-binding potential of LMPH-derived peptides and highlight their future potential as functional ingredients for preventing and managing iron deficiency.

## 1. Introduction

Iron is an essential micronutrient for maintaining human metabolism, and its deficiency is associated with various health disorders [1]. Several strategies have been developed to address this issue, mainly through supplementation with ferrous salts [2,3]. However, their application and effectiveness are limited by adverse effects (such as gastrointestinal irritation, nausea and vomiting, constipation, among others) as well as by problems related to sensory acceptability and stability in food matrices [4]. In this context, the development of bioavailable iron fortifiers compatible with food products has become a major challenge in food science and technology [5].

Iron chelation with bioactive peptides has been proposed as one of the most promising strategies due to its high intestinal absorption, adequate bioavailability, favourable safety profile, and greater stability in food systems [6]. Accordingly, peptide–iron chelates represent innovative functional supplements with advantages over conventional iron formulations [7]. Currently, numerous studies have evaluated peptides derived from food protein matrices for their metal-binding properties [8,9,10]. Plant proteins have been extensively investigated as sustainable sources of metal-chelating peptides. Their production generally involves a sequence of steps including enzymatic hydrolysis, separation, concentration, and purification, followed by identification using LC-MS/MS and validation through *in vitro*, *in silico*, and *in vivo* approaches [7,11,12].

Several studies have described common structural features of peptides exhibiting affinity for iron. These include a high content of amino acids with functional groups capable of coordinating metals, such as Glu (E) and Asp (D), which interact through carboxylate oxygen atoms with the vacant orbitals of ferrous ions [8,13]. Histidine (H) can also donate electron pairs via the nitrogen atoms of its imidazole ring. Additionally, Cys (C) and Ser (S) participate in iron binding through their thiol (–SH) and hydroxyl (–OH) groups, respectively. Other functional groups, such as the guanidino group of Arg (R) and the ε-amino group of Lys (K), contribute to complex stabilization [14,15]. Another relevant characteristic is the low molecular weight of these peptides (≤1500 Da, approximately 2–10 residues), which increases the proportion of free amino and carboxyl groups, thereby enhancing interactions with metal ions [16]. Moreover, the presence of multiple binding sites improves chelation stability through the formation of internal coordination rings. For instance, –Glu–Glu– motifs may act as effective multidentate ligands. Finally, the conformational flexibility of peptides enables the proper orientation of functional groups around Fe^2+^, favouring the formation of stable complexes [17].

*Lupinus mutabilis*, commonly known as “tarwi,” is a legume characterized by its high protein content (44% dry weight), which exceeds that of other species of the *Lupinus* genus, such as *Lupinus albus*, *Lupinus luteus*, and *Lupinus angustifolius* [18]. Furthermore, it exhibits an amino acid profile rich in glutamic acid (24.3 g/100 g protein) and aspartic acid (9.6 g/100 g protein), which are closely associated with high iron-chelating capacity [18]. In this context, *Lupinus mutabilis* emerges as a promising source of peptides with iron-chelating potential. Thus, the objectives of this study were to: (1) obtain protein hydrolysates from *Lupinus mutabilis*; (2) concentrate, purify, and identify iron-chelating peptides derived from these hydrolysates; and (3) evaluate their iron-chelating potential using *in silico* bioinformatics tools.

## 2. Materials and Methods

### 2.1. Materials and Reagents

*Lupinus mutabilis* (LM) seeds were obtained from the Programa de Leguminosas y Oleaginosas of the Universidad Nacional Agraria La Molina (UNALM, Lima, Peru). Alcalase^®^ enzyme (2.4 AU/g), ferrous sulphate heptahydrate (FeSO_4_·7H_2_O, 99.5%), IDA–Sepharose^®^ resin, and ferrozine [3-(2-pyridyl)-5,6-diphenyl-1,2,4-triazine-4,4′-disulfonate sodium salt] were purchased from Sigma-Aldrich (St. Louis, MO, USA). All other reagents used in this study were of analytical or chromatographic grade and were obtained from Merck (Darmstadt, Germany), Sigma-Aldrich (St. Louis, MO, USA), or J.T. Baker (Phillipsburg, NJ, USA).

### 2.2. Preparation of Lupinus mutabilis Peptides

#### 2.2.1. Protein Extraction

The LM grains were subjected to a debittering process by continuous washing with distilled water, using a water-to-grain ratio of 5:1 (*v*/*w*) for 24 h. The washing water was replaced after 6, 12, and 24 h. Subsequently, the grains were dried and milled to obtain a particle size ranging from 0.5 to 1.0 mm. Defatting of the flour was carried out under continuous stirring using petroleum ether at a ratio of 3:1 (solvent:flour, *v*/*w*). The recovered solid was dried at 35–40 °C to remove residual solvent and was referred to as debittered and defatted LM flour (DDLMF). Thereafter, the protein concentrate (protein content: 76.6%) was obtained from DDLMF following the methodology described by Chirinos et al. [19].

#### 2.2.2. Preparation and Concentration of the Protein Hydrolysate

The protein hydrolysate was obtained from the protein concentrate as follows. The concentrate was dispersed in deionized water to a final protein concentration of 2.5% (*w*/*v*). The pH of the suspension was adjusted and maintained at 8.2 by the addition of 1 N NaOH before and during enzymatic hydrolysis. Subsequently, Alcalase^®^ was added at an enzyme-to-substrate ratio of 0.385 AU per gram of protein. The reaction was carried out under constant agitation at 50 °C and maintained for 180 min. Enzyme inactivation was achieved by heating at 100 °C for 10 min. The reaction mixture was then cooled to room temperature and centrifuged at 16,270× *g* for 15 min at 4 °C. The resulting supernatant was filtered through a 0.45 μm membrane filter, and the final product was designated as *Lupinus mutabilis* protein hydrolysate (LMPH).

LMPH was sequentially fractionated using a batch-mode ultrafiltration system equipped with Hydrosart^®^ membranes (Sartorius, Göttingen, Germany) with molecular weight cut-offs (MWCO) of 10 and 2 kDa at room temperature. The following fractions were collected and stored: >10 kDa (UF >10 kDa), 2–10 kDa (UF 2–10 kDa), and <2 kDa (UF < 2 kDa).

### 2.3. Immobilized Metal Affinity Chromatography

Iron-chelating peptides were isolated using immobilized metal affinity chromatography (IMAC), following the methodology described by Guo et al. [20] with minor modifications. Briefly, a glass column (15 mm × 200 mm) was packed with 20 mL of iminodiacetic acid Sepharose^®^ resin (IDA–Sepharose) purchased from Sigma-Aldrich (St. Louis, MO, USA). The column was connected to a BioLogic LP system and a BioFrac fraction collector (Bio-Rad). Prior to metal loading, the column was equilibrated with six bed volumes of Milli-Q water. Subsequently, the matrix was charged with a 0.2 mol·L^−1^ Fe^2+^ solution prepared from FeSO_4_·7H_2_O. Unbound iron was removed by washing the column with three bed volumes of Milli-Q water. Then, 2 mL of sample at a concentration of 45 mg protein·mL^−1^ (from the UF < 2 kDa fraction), prepared in Milli-Q water and adjusted to pH 7.6, was injected and allowed to interact with the matrix overnight at room temperature. Unretained peptides were removed by washing the column with the same solvent until the absorbance, monitored at 214 nm, returned to baseline. Bound peptides were eluted using Milli-Q water adjusted to pH 3.0. Once the absorbance at 214 nm returned to baseline, a final elution step was performed at pH 2.0. The flow rate was maintained at 1 mL·min^−1^ throughout the procedure (sample loading and elution). Eluates were pooled according to the chromatographic profile (based on absorbance measurements), yielding well-resolved fractions (fractions F1 to F3). Soluble protein content and Fe^2+^-chelating capacity were determined for each fraction. The selected fractions were subsequently lyophilized for further analysis. After elution, the column was washed and regenerated using a 50 mmol·L^−1^ EDTA solution.

### 2.4. Gel Filtration Chromatography

Gel filtration chromatography (GFC) was performed using a glass column (10 mm × 1000 mm) packed with Bio-Gel^®^ P-2 resin (Bio-Rad Inc., Hercules, CA, USA). The column was connected to the BioLogic LP system and the BioFrac fraction collector (Bio-Rad). Prior to packing, the resin was hydrated and equilibrated with 3% (*v*/*v*) methanol solution, following the methodology described by Chirinos et al. [21]. Lyophilized fractions obtained from IMAC–Fe^2+^ chromatography were resuspended in Milli-Q water (~45 mg·mL^−1^ and ~70 mg·mL^−1^ for fractions F2 and F3, respectively) and loaded onto the column. Elution was monitored at 214 nm. The flow rate was maintained at a constant flow rate of 0.2 mL·min^−1^ throughout the procedure. Eluates were pooled according to the chromatographic profile (based on absorbance measurements) yielding well-separated subfractions. Thus, from F2 and F3 fractions a total of six (F2A–F2F) and three sub-fractions (F3A–F3C) were obtained. Soluble protein content and Fe^2+^-chelating capacity were determined for each subfraction. Subfractions exhibiting the highest iron-chelating capacity were selected for peptide identification by LC-MS/MS.

### 2.5. Identification of Lupinus mutabilis Peptides by LC-MS/MS

The freeze-dried peptides were reduced with dithiothreitol (~55 °C for 45 min) and alkylated with iodoacetamide under dark conditions (at room temperature for 30 min) to ensure accurate structural characterization. Subsequently, samples were desalted to remove salts and other impurities using C18 spin columns and eluted with acetonitrile in the presence of formic acid prior to LC-MS/MS analysis. Peptides were separated via the EVOSEP^®^ 60 SPD method and analyzed in DDA mode with the Zenotof (ABSCIEX). The EVOSEP^®^ 60 SPD method is a standardized LC-MS workflow and was performed according to the manufacturer’s instructions. The 60 SPD method has a 21 min gradient and a cycle time of 24 min. The analytical column was equilibrated at 2 μL·min^−1^. The gradient flow was 1 μL·min^−1^ and increased to 2 μL·min^−1^ for washing. Raw data were loaded in Progenesis QI for proteomics (non-linear dynamics). MGF files were exported and send for analysis to Novor [22]. The spectra were searched against (i) the Novor Swissprot database, (ii) a custom-made fraction of the Uniprot database containing all known proteins from the *Lupinus* genus (taxonomy ID 3869) (target plus reversed decoy accessions = 113,282) and (iii) a *de novo* sequencing was performed. In addition, identified peptides were evaluated *in silico* for their metal-chelating capacity using AnOxPePred-1.0 plataform (https://services.healthtech.dtu.dk/services/AnOxPePred-1.0/ (accessed on 7 January 2026)) where the predictive values range from 0 to 1, with 0 indicating no chelating activity and 1 indicating a high probability of chelating activity. In addition, the possibly toxicity was evaluated with ToxinPred 3.0 tool (https://webs.iiitd.edu.in/raghava/toxinpred/multi_submit.php (accessed on 7 January 2026)).

### 2.6. Molecular Docking of Lupinus mutabilis Peptides with Fe^2+^-Chelating Capacity

#### 2.6.1. Receptor and Ligand Preparation

Docking simulations were performed on a subset of peptides selected based on three criteria: (i) origin from the most bioactive fractions (F2D and F3C), (ii) presence of amino acid residues commonly associated with metal binding (Asp, Glu, His, Cys), and (iii) positive chelation prediction using AnOxPePred combined with absence of predicted toxicity.

The peptide receptors investigated were manually built using Discovery Studio Visualizer (BIOVIA, Dassault Systèmes, San Diego, CA, USA). The resulting three-dimensional structures were then subjected to geometry optimization employing Avogadro software (version 1.2.0) [23]. Structural relaxation was performed through the Auto Optimization Tool, using the Universal Force Field (UFF) with four steps per update and the steepest descent algorithm. The minimization process was considered converged when the energy difference between successive steps was lower than 0.01 kJ·mol^−1^. After optimization, all peptide structures were saved in PDB format for subsequent docking preparation. The Fe^2+^ ion used as ligand was obtained from PubChem (CID: 27284) in SDF format. The structure was converted to PDB and subsequently to PDBQT format using AutoDockTools [24], following the standard preparation protocol required for docking simulations.

#### 2.6.2. Docking Protocol and Scoring Analysis

Each optimized peptide receptor was imported into AutoDockTools (MGLTools version 1.5.7) for docking setup. Receptor-specific grid boxes were individually defined based on the spatial dimensions of each peptide, ensuring full coverage of the molecular surface and allowing unrestricted exploration of potential interaction regions by the ligand during the docking procedure.

Docking calculations were performed using AutoDock 4 [25], employing the Lamarckian Genetic Algorithm (LGA) as the search strategy, combined with a semi-empirical free energy force field to explore ligand conformational space. The algorithm iteratively searches for optimal binding poses through a hybrid global and local search strategy, while the scoring function estimates binding energies based on intermolecular contributions such as van der Waals interactions, hydrogen bonding, electrostatics, and desolvation effects. Default docking parameters recommended for small-molecule ligands were applied, with modifications restricted to the grid dimensions tailored for each peptide receptor. Multiple docking runs were conducted for each receptor–ligand system, and the resulting conformations were ranked according to AutoDock’s semi-empirical free-energy scoring function. The highest-ranked poses were subsequently subjected to visual inspection and structural analysis using PyMOL (version 3.0) (Schrödinger, LLC, New York, NY, USA). This analysis focused on evaluating metal–peptide spatial relationships, including minimum distances between Fe^2+^ and peptide residues, as well as on generating high-quality molecular representations of the predicted complexes. Therefore, the docking results should not be interpreted as representing true coordination complexes, but rather as indicative of potential interaction regions.

### 2.7. Soluble Protein

The soluble protein content was measured using the Lowry method with bovine serum albumin (BSA) as the standard according to the methodology proposed by Chirinos et al. [19]. Protein hydrolysate samples were reacted with alkaline copper reagent, followed by the Folin–Ciocalteau reagent, and incubated to develop colour. Absorbance was read at 750 nm, and protein concentrations were calculated from a BSA calibration curve (0.01–0.5 mg·mL^−1^) and reported as mg·mL^−1^.

### 2.8. Degree of Hydrolysis

The degree of hydrolysis (DH) was determined by quantifying free amino groups using the 2,4,6-trinitrobenzenesulfonic acid (TNBS) method, according to the procedure described by Adler-Nissen [26]. Quantification was performed using L-leucine as the standard, and absorbance was measured at 340 nm based on a calibration curve of leucine (0.6–3.0 mM).

The total amino group content in the *Lupinus mutabilis* protein hydrolysate (LMPH) was determined after complete acid hydrolysis using 6 M HCl at 120 °C for 24 h. The DH was expressed as the percentage of hydrolysed peptide bonds and calculated as the ratio between free amino groups in LMPH and the total amino groups obtained after complete acid hydrolysis, as follows:

DH (%) = [(AN_2_ − AN_1_)/ Npb] ∗ 100

where AN_1_ represents the amino nitrogen content in the protein before hydrolysis, AN_2_ represents the amino nitrogen content after enzymatic hydrolysis, and Npb corresponds to the amino nitrogen of peptide bonds in the protein sample after complete acid hydrolysis.

### 2.9. Determination of Iron-Chelating Capacity

Iron-chelating capacity (Fe^2+^) (ICC) was determined according to the method described by Arise et al. [27] with minor modifications. LMPH, ultrafiltered fractions, and peptide fractions/subfractions obtained from IMAC–Fe^2+^ and GFC were adjusted to a final soluble protein concentration of 1 mg·mL^−1^. Subsequently, samples were mixed with 0.05 mL of FeSO_4_·7H_2_O (2 mM) and 1.85 mL of Milli-Q water in reaction tubes and incubated at room temperature for 1 h. After incubation, 0.1 mL of ferrozine (5 mM) was added and gently mixed. The reaction was allowed to proceed at room temperature for 10 min. Then, 200 μL of the reaction mixture was transferred to a 96-well microplate, and absorbance was measured at 562 nm using a microplate reader (BioTek Instruments Eon, Winooski, VT, USA). The control was prepared by replacing the sample with Milli-Q water. Results were expressed as ICC (mg Fe^2+^·g^−1^ protein) and as iron-chelating rate (%), using the following equations:

$$ICC=\frac{M_{1}-M_{2}}{M_{0}}$$

where M_1_ corresponds to the total mass of iron (mg) added as ferrous salt, M_2_ represents the total mass of unchelated ferrous ions, and M_0_ is the mass of added peptides (g).

The iron-chelating rate was calculated as:

$$Iron\;chelating\;rate=\frac{A_{b}-A_{s}}{A_{b}}*100$$

where A_b_ and A_s_ represent the absorbance values of the control and sample, respectively.

### 2.10. Statistical Analysis

Results were expressed as mean ± standard deviation. Data were analysed using one-way analysis of variance (ANOVA), followed by Tukey’s post hoc test. Differences were considered statistically significant at *p* < 0.05. Statistical analyses were performed using R software (version 4.5.0).

## 3. Results and Discussion

### 3.1. Production, Characterization and Iron-Chelating Capacity of Lupinus mutabilis Protein Hydrolysate and Its Fractions

The DH allows structural differentiation among protein hydrolysates and represents an important parameter influencing their functional properties [28]. One of these functional properties is the ability to chelate divalent metal ions, such as Fe^2+^, which is favoured by high DH values due to the generation of low-molecular-weight peptides from parent proteins [9]. The LMPH obtained in this study reached a DH of 45.51 ± 0.04%, indicating extensive hydrolysis and suggesting the predominance of low-molecular-weight peptides with potential metal-chelating properties [8,29]. The DH value obtained was very close to that reported by Chirinos et al. [19] (41.1%) for the same raw material subjected to identical hydrolysis conditions with Alcalase^®^, confirming the reproducibility of the enzymatic hydrolysis process.

The ICC value (22.75 ± 0.61 mg Fe^2+^·g^−1^ protein) and iron-chelating rate (55 ± 0.03%) observed for LMPH suggest that enzymatic hydrolysis generated peptides with affinity for Fe^2+^. This behavior is explained by their low molecular weight, which enhances structural flexibility and increases the exposure of functional groups available for metal coordination, as well as by the presence of amino acid residues capable of forming coordination bonds or other interactions with metal ions (e.g., Fe^2+^) [9,10]. The ICC values obtained were higher than those reported for *Acetes japonicus* hydrolysates produced using Flavourzyme (0.13 mg Fe^2+^·g^−1^ protein) but lower than those reported for whey protein hydrolysates (36.42 ± 0.184 mg Fe^2+^·g^−1^ protein) [12,30]. Regarding iron-chelating rate, the value obtained in this study was higher than that reported for other hydrolysates, such as green bean, Pacific cod skin, and abalone viscera protein hydrolysates (50.2, 12.7, and 16.2%, respectively) [12,30,31,32].

To concentrate and purify peptides associated with metal-chelating activity, LMPH was subjected to sequential ultrafiltration using 10 and 2 kDa membranes, a technique widely applied for the fractionation of bioactive peptides [33]. This process yielded three fractions: UF > 10 kDa, UF 2–10 kDa, and UF < 2 kDa (Table 1).

The UF < 2 kDa fraction exhibited the highest ICC (35.16 ± 0.99 mg Fe^2+^·g^−1^ protein), representing a ~55% increase compared with LMPH. In addition, this fraction showed a high iron-chelating rate (69.40 ± 2.41%), significantly surpassing not only LMPH but also all other fractions obtained. These results are consistent with previous studies that employed ultrafiltration membranes < 2–3 kDa as an effective strategy for concentrating iron-chelating peptides [10,30,34]. Previous studies have associated low-molecular-weight peptides with higher affinity for metal ions, which is mainly attributed to reduced steric hindrance and increased accessibility of metal-binding sites [34]. Overall, these results indicate that enzymatic hydrolysis of *Lupinus mutabilis* protein concentrate followed by ultrafiltration favours the enrichment of low-molecular-weight peptides with high ICC. Therefore, the UF < 2 kDa fraction emerges as one of the most promising candidates for application as a functional ingredient in food systems.

### 3.2. Separation of Fe^2+^-Binding Peptides Using Chromatographic Techniques

**Figure 1:**
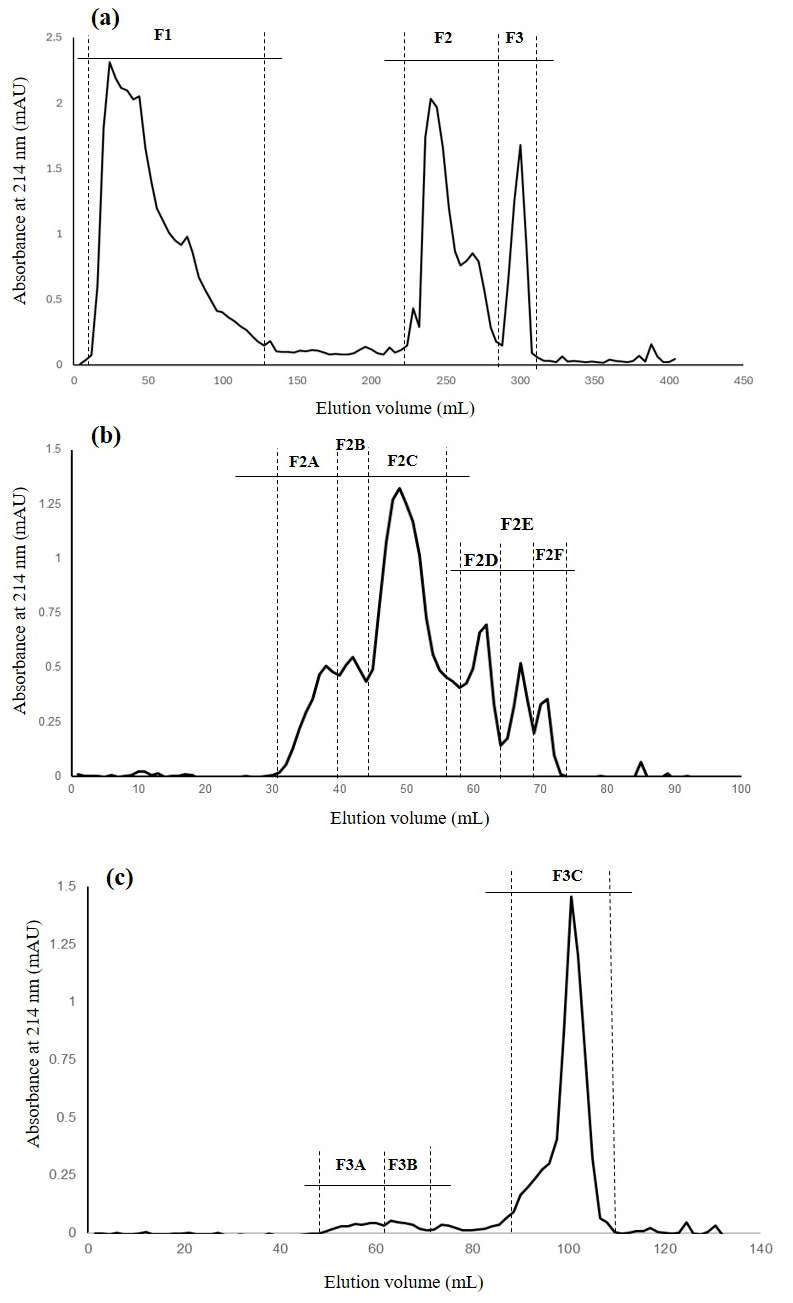
(**a**) Chromatographic profile (IMAC–Fe^2+^) of the UF < 2 kDa fraction; (**b**) gel filtration chromatography profile of F2 obtained from IMAC (dead and inclusion volume: 30 and 74 mL, respectively); and (**c**) gel filtration chromatography profile of F3 obtained from IMAC (dead and inclusion volume: 45 and 112 mL, respectively).

The UF < 2 kDa fraction was further purified using two chromatographic systems, IMAC–Fe^2+^ followed by GFC, to isolate and identify peptides capable of chelating divalent iron (Figure 1). 

IMAC is commonly applied as an initial step in the separation of metal-chelating peptides. The principle of this technique is based on the interaction between biomolecules (peptides or proteins) and immobilized metal ions (in this case Fe^2+^), which occurs according to Lewis acid–base theory [33]. Accordingly, the IMAC chromatogram obtained (Figure 1a) was consistent with previous reports [35,36]. In the present study, three well-defined fractions (F1–F3) were obtained.

The first fraction (F1) corresponded to the unretained fraction and was mainly composed of peptides exhibiting weak or nonspecific interactions with iron [37], as reflected by its low ICC (13.07 ± 0.60 mg Fe^2+^·g^−1^ protein) value. In contrast, the subsequently eluted fractions (F2 and F3) were enriched in peptides with higher affinity for Fe^2+^. Fraction F2 exhibited the highest ICC value (51.06 ± 0.19 mg Fe^2+^·g^−1^ protein), followed by F3 (33.33 ± 9.91 mg Fe^2+^·g^−1^ protein; Table 2). A similar trend was observed for iron-chelating rates. These values were significantly higher (*p* < 0.05) than those obtained for the initial hydrolysate and the UF < 2 kDa fraction, indicating the high selectivity of the IMAC system for recovering iron-chelating peptides.

The differential retention of peptides on the IMAC matrix may be attributed to the formation of coordination bonds between peptide functional groups and iron ions, as well as to electrostatic, hydrophobic, and van der Waals interactions. The loading pH (7.6) may have favoured partial oxidation of Fe^2+^ to Fe^3+^, thereby modifying the behaviour of the immobilized metal and its interaction mode with peptides. Under these conditions, iron tends to behave as a strong Lewis acid, showing preferential binding to acidic residues such as Asp (D) and Glu (E) rather than to His (H), as previously described for iron-charged IMAC matrices [36,38]. This is consistent with studies reporting iron-chelating peptides enriched in Asp and Glu, even in the absence of His residues [9,39], while also suggesting a possible contribution of other polar amino acids such as Ser (S) and Thr (T) [37].

Given the high ICC values of fractions F2 and F3, they were independently subjected to GFC as an additional purification step prior to peptide sequence identification by LC-MS/MS. GFC separates molecules based on their size-dependent migration rates rather than physicochemical properties, with larger molecules eluting faster than smaller ones [40]. From fraction F2, six subfractions (F2A–F2F) were obtained as shown in Figure 1b and Table 3. 

Notably, subfraction F2D exhibited the highest ICC value (45.20 ± 0.40 mg Fe^2+^·g^−1^ protein) and the greatest iron-chelating rate (87.25 ± 0.50%). Similarly, from fraction F3, three subfractions (F3A–F3C) were obtained as shown in Figure 1c. The first two (F3A and F3B) were recovered in small amounts, whereas subfraction F3C was predominant and displayed an iron-chelating capacity of 13.51 ± 0.44 mg Fe^2+^·g^−1^ protein and a chelation rate of 6.0 ± 0.81%. These values are comparable to those reported by Ding et al. [41], who obtained five peptide fractions from Sephadex G-15 chromatography of mung bean hydrolysates, with ICC values ranging from approximately 27 to 39.9 μg·mg^−1^. Peptide fractions exhibiting high ICC are generally associated with the presence of low-molecular-weight peptides with strong metal-binding affinity, supported by favourable amino acid composition and structural conformation [11,15].

### 3.3. Peptide Identification by LC-MS/MS De Novo Sequencing

To date, comprehensive databases of sequenced proteins from *Lupinus mutabilis*, particularly conglutin-type proteins (β-, α-, γ-, and δ-conglutins), which are considered the most abundant storage proteins in the *Lupinus* genus, remain limited. This limitation supports the use of LC-MS/MS *de novo* sequencing for peptide identification in the selected subfractions obtained in the previous step. Accordingly, a total of 126 and 50 peptides with *Novor scores* higher than 85 were identified from subfractions F2D and F3C, respectively. Among these, several sequences showed high similarity or close homology to proteins from *Lupinus angustifolius*, such as conglutin β1 and β2, uncharacterized proteins, and conglutin α1 (Supplementary Tables S1 and S2).

A total of 176 peptides were identified by LC-MS/MS analysis from sub-fractions F2D and F3C. Peptides with the highest potential for iron chelation were selected according to the following structure–ICC property considerations: (1) metal binding may occur through amino- and carboxyl-terminal groups, peptide bonds, and amino acid side chains. In this context, Asp (D), Glu (E), His (H), Cys (C), Ser (S), and Arg (R) have been reported to exhibit iron-chelating activity, with the first four showing the highest affinity [15,42]; (2) Asn (N) and Gln (Q) contribute to the formation of thermodynamically stable peptide–metal complexes, although their amide groups are generally considered non-coordinating [43]; and (3) short oligopeptides (2–10 residues) facilitate accessibility and flexibility of chelating sites. Thus, based on these criteria, peptides containing at least two residues of type D, E, H, and/or C (alone or in combination), possibly accompanied by one or more S or R residues, as well as N and Q residues, were selected. In addition, peptides were required to contain a minimum of three residues from the above-mentioned amino acids. Under these criteria, a total of 17 and 12 peptides were selected from subfractions F2D and F3C, respectively (Table 4). 

The peptides SNEPLYR and RYDRDGQLR were common to both subfractions. In all cases, peptide length ranged from 3 to 10 amino acid residues. Furthermore, the metal-chelating capacity of the selected peptides was predicted using the AnOxPePred 1.0 platform [44]. All peptides exhibited predicted chelating activity, with values ranging from 0.214 to 0.275 (Table 4). These values are comparable to those reported by Zhang et al. [45], who found chelation scores between 0.1721 and 0.2922 in antioxidant peptides derived from fermented sheep milk. In addition, none of the evaluated *Lupinus mutabilis* peptides showed potential toxicity.

Thus, peptides derived from sub-fractions F2D and F3C (29 in total; Table 4) that met the selection criteria—namely, the presence of residues associated with iron binding (e.g., D, E, H, C, among others described in the previous paragraph) and a positive chelation prediction (AnOxPePred) with no predicted toxicity—corresponded to a set of candidate structures. Specifically, the following peptides (9 in total) were identified and selected: RYDRDGQLR, QQPLPR, YDFLHF, SNEPLYR, FDGWQPR, and EDYRFY from sub-fraction F2D, and EPR, SNEPLYR, SPPTLRPR, RYDRDGQLR, and REPSLR from sub-fraction F3C. These candidates were selected for further evaluation through molecular docking analysis.

### 3.4. Molecular Docking Analysis of Identified Peptides from Lupinus mutabilis

Molecular docking analysis suggested differences in predicted interaction patterns between Fe^2+^ and the evaluated peptides. The interpretation of docking results considers the estimated binding energies based on intermolecular contributions such as van der Waals interactions, hydrogen bonding, electrostatics, and desolvation. As these contributions are primarily parameterized for organic ligand–receptor systems, the obtained binding energy values should be interpreted with caution. Thus, binding energy values were not interpreted as absolute affinity measures, but only as relative indicators, due to the known limitations of AutoDock4 scoring functions for metal ion systems. Therefore, in this study, greater emphasis was placed on spatial interaction patterns, such as residue proximity and metal–peptide distances, rather than on absolute energy values.

Structurally, the estimated interaction profile was reflected in docking poses in which Fe^2+^ remained predominantly external to the peptide framework (Table 5). A first subset of peptides exhibited large metal–peptide separations. This behaviour was observed for RYDRDGQLR and REPSLR, in which Fe^2+^ remained at the periphery of the molecular system and did not explore cavities or semi-enclosed regions formed by the peptide backbone. A second group displayed intermediate Fe^2+^–peptide distances (approximately 4.5–5.9 Å), suggestive of preferential regions of transient proximity but incompatible with direct coordination. This group included EPR, SPPTLRPR, and YDFLHF. A third group comprised peptides exhibiting shorter Fe^2+^–peptide distances, indicating localized sites of preferential approach. Peptides containing acidic residues such as Asp and Glu exhibited shorter Fe^2+^–residue distances, which is consistent with known coordination preferences of iron ions. The most notable case was EDYRFY, in which Fe^2+^ was positioned at approximately 2.9 Å from the carboxylate group of Glu1, suggesting close spatial proximity compatible with electrostatic interaction. The results further suggested that aromatic residues may act as recurrent regions of transient Fe^2+^ proximity. In SNEPLYR, Fe^2+^ approached Tyr6 at approximately 3.8 Å, a distance compatible with weak cation–π or π-polarization interactions, although the positioning appeared peripheral rather than centrally aligned over the aromatic ring. In contrast, FDGWQPR exhibited a larger separation from Phe1 (~5.6 Å), rendering any cation–π contribution marginal. These observations indicate that exposed aromatic environments may increase the likelihood of transient metal proximity. The QQPLPR highlighted a recurring tendency for Fe^2+^ to approach conformationally constrained or hydrophobic regions, particularly those involving Pro residues. The rigidity imposed by proline may favour the formation of local surfaces that are more frequently sampled by metal ions. The external positioning of Fe^2+^ evidenced from the analysis is attributed to the small size and flexibility of the peptides, which do not form well-defined binding pockets.

Finally, the predicted binding energies within a narrow range (approximately −0.42 to −0.74 kcal·mol^−1^) were found, which are comparable to values reported for iron-binding peptides derived from whey protein (−1.2 to −0.6 kcal·mol^−1^) [46] and from *Amaranthus* globulin protein (−0.624 and −0.634 kcal·mol^−1^) [47].

Based on the previously described findings, these interaction patterns provide a solid basis for understanding iron affinity *in vitro*, serving as an important predictive step that signals the tendency for complex formation. While final stability depends on dynamic factors beyond the scope of this static model, these results effectively guide ongoing investigations. Further studies on the formation of complexes between *Lupinus mutabilis* protein hydrolysate-derived peptides and Fe^2+^ and their stability under gastrointestinal digestion conditions are currently underway.

## 4. Conclusions

Sequential ultrafiltration using 10 and 2 kDa membranes promoted the enrichment of low-molecular-weight peptides with iron-chelating capacity, yielding higher activity than that observed in the original *Lupinus mutabilis* protein hydrolysates. The combined application of IMAC and gel filtration chromatography enabled the effective enrichment of iron-affinity peptides, establishing this approach as a suitable strategy prior to peptide identification. LC-MS/MS analysis allowed the identification of a wide range of peptides, including novel sequences and others showing similarity to proteins from *Lupinus angustifolius*. Based on structure–property relationships associated with metal chelation, nine peptides were selected for molecular docking analysis. Docking studies indicated potential interaction regions between Fe^2+^ and the selected peptides. Overall, the results indicate that *Lupinus mutabilis* protein hydrolysates subjected to ultrafiltration with a molecular weight cut-off of <2 kDa contain a substantial proportion of peptides capable of interacting with Fe^2+^. This positions these hydrolysates as promising candidates for the development of peptide–iron complexes as functional ingredients, with potential application as iron fortifiers for the prevention and management of iron deficiency. Based on the results obtained, further studies are required to evaluate the feasibility of scaling up production, the sensory impact of incorporating these peptides into food matrices, and to assess their stability, bioaccessibility, and bioavailability (e.g., Caco-2 cell uptake studies and *in vivo* trials) in order to validate their nutritional functionality.

## Figures and Tables

**Table 1:** Iron chelation capacity and iron chelation rate evaluated in the protein hydrolysate and the different fractions obtained after the ultrafiltration process.

Fraction	Iron Chelation Capacity (mg Fe^2+^·g^−1^ Protein) *	Rate of Iron Chelation (%) *
LMPH	22.75 ± 0.61 ^c^	54.67 ± 1.15 ^bc^
UF > 10 kDa	26.19 ± 0.36 ^b^	58.33 ± 0.58 ^b^
UF < 10 kDa –> 2 kDa	18.21 ± 0.61 ^d^	44.33 ± 1.53 ^c^
UF < 2 kDa	35.16 ± 0.99 ^a^	69.40 ± 2.41 ^a^

* Mean of three replicates ± SD. Mean values with different letters within the same column indicate significant differences according to Tukey’s test (*p* < 0.05).

**Table 2:** Iron chelation capacity and iron chelation rate of the fractions obtained by IMAC.

Fraction IMAC	Iron Chelation Capacity (mg Fe^2+^·g^−1^ Protein) *	Rate of Iron Chelation (%) *
F1	13.07 ± 0.60 ^c^	N.D
F2	51.06 ± 0.19 ^a^	94.75 ± 0.50 ^a^
F3	33.33 ± 9.91 ^b^	71.25 ± 2.36 ^c^

* Mean of three replicates ± SD. Mean values with different letters within the same column indicate significant differences according to Tukey’s test (*p* < 0.05). N.D: Not detected.

**Table 3:** Iron-chelating capacity and iron chelation rate of the F2 subfraction from IMAC and its subsequent gel filtration chromatography.

Fraction	Iron Chelation Capacity (mg Fe^2+^·g^−1^ Protein) *	Rate of Iron Chelation (%) *
F2A	N.D	N.D
F2B	0.77 ± 0.06 ^d^	1.00 ± 0.01 ^d^
F2C	11.96 ± 0.12 ^b^	5.25 ± 0.50 ^b^
F2D	45.20 ± 0.40 ^a^	87.25 ± 0.50 ^a^
F2E	1.86 ± 0.16 ^d^	7.00 ± 0.00 ^d^
F2F	8.36 ± 1.42 ^c^	14.50 ± 4.04 ^c^

* Mean of three replicates ± SD. Mean values with different letters within the same column indicate significant differences according to Tukey’s test (*p* < 0.05). N.D: Not detected.

**Table 4:** Peptides from *L. mutabilis* with metal-chelating affinity identified by LC-MS/MS *de novo* sequencing.

N°	*m*/*z*	*z*	Score	Peptide Mass	Error (ppm)	Length	*De Novo* Peptide	Chelating Capacity ^&^	Toxicity ^§^
	Peptides from subfraction F2-E								
1	453.21	2	96.2	904.41	2.6	7	FDGWQPR	0.2518	Non-Toxin
2	420.20	2	95.9	838.38	2.4	6	DWYDLK	0.2143	Non-Toxin
3	446.69	2	95.9	891.37	2	6	EDYRFY	0.249	Non-Toxin
4	386.69	2	95.4	771.36	2.1	6	EGWQPR	0.2411	Non-Toxin
5	415.20	2	94.8	828.38	4.3	7	ADGWQPR	0.2400	Non-Toxin
6	429.21	2	94.7	856.41	5.5	7	VDGWQPR	0.2299	Non-Toxin
7	439.72	2	93.9	877.42	2	7	SNEPLYR **	0.2530	Non-Toxin
8	422.21	2	93.6	842.40	7.5	7	AEGWQPR	0.2362	Non-Toxin
9	386.69	2	93.4	771.36	2.1	6	ADWQPR	0.2452	Non-Toxin
10	369.71	2	92.5	737.41	−0.2	6	QQPLPR *	0.2730	Non-Toxin
11	375.16	2	91.6	748.31	1.7	5	DWDKW	0.2160	Non-Toxin
12	461.21	2	91.2	920.41	2.1	7	YDGWQPR	0.2427	Non-Toxin
13	354.68	2	91	707.36	−0.5	5	EYLRQ	0.2337	Non-Toxin
14	421.19	2	89.6	840.38	1	6	YDFLHF	0.2687	Non-Toxin
15	415.20	2	89.4	828.38	4.3	6	ENWQPR	0.2440	Non-Toxin
16	393.53	3	88.3	1177.59	1.9	9	RYDRDGQLR **	0.2758	Non-Toxin
17	460.22	2	87.7	918.43	8.4	7	FEGWQPR	0.2481	Non-Toxin
	Peptides from subfraction F3-C								
1	364.70	2	96.7	727.39	1.9	6	VNPDKRQ *	0.2244	Non-Toxin
2	421.24	2	95.8	840.48	1.2	7	LVNPDKR *	0.2498	Non-Toxin
3	401.21	1	94.5	400.20	1.7	3	EPR	0.2749	Non-Toxin
4	439.72	2	94	877.42	2	7	SNEPLYR **	0.2536	Non-Toxin
5	462.27	2	91	922.53	0.5	8	SPPTLRPR *	0.2567	Non-Toxin
6	361.19	2	90.4	720.36	3.1	5	RFDQR *	0.2320	Non-Toxin
7	353.68	2	90.2	705.35	1.8	5	RDYPR	0.2145	Non-Toxin
8	415.20	2	88.9	828.38	4.3	7	DAGWQPR	0.2466	Non-Toxin
9	379.23	3	88.1	1134.68	−0.5	10	VLSPPTLRPR *	0.2432	Non-Toxin
10	393.53	3	85.7	1177.59	1.9	9	RYDRDGQLR **	0.2758	Non-Toxin
11	379.21	2	85.4	756.42	0.3	6	REPSLR	0.2690	Non-Toxin
12	478.28	2	85. 3	954.54	7.8	7	RDKRQPR	0.2159	Non-Toxin

(*) Peptides identified derived from *Lupinus angustifolius* proteins. (**) Peptides found in both evaluated subfractions. ^&^ Data accessed from AnOxPePred-1.0 (https://services.healthtech.dtu.dk/services/AnOxPePred-1.0/). ^§^ Potential toxicity was obtained from ToxinPred 3.0 (https://webs.iiitd.edu.in/raghava/toxinpred/multi_submit.php). All databases were consulted in 7 January 2026.

**Table 5:** Molecular docking analysis of peptides identified in *Lupinus mutabilis*.

N°	Peptide	Binding Energy (kcal/mol)	Residue Closest/Distance	Spatial Proximity of the Peptide to Fe^2+^
1	FDGWQPR	−0.63	Phe 1 5.6 Å	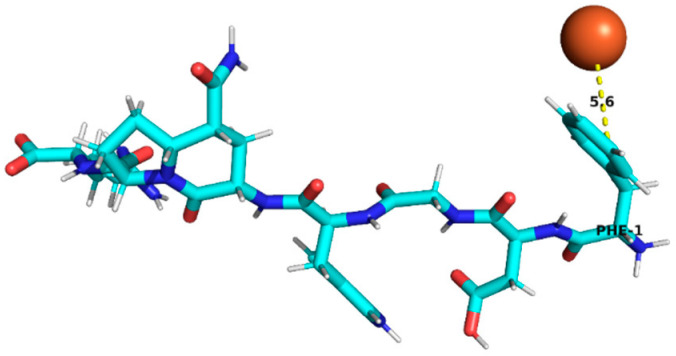
2	EDYRFY	−0.63	Glu 1 2.9 Å	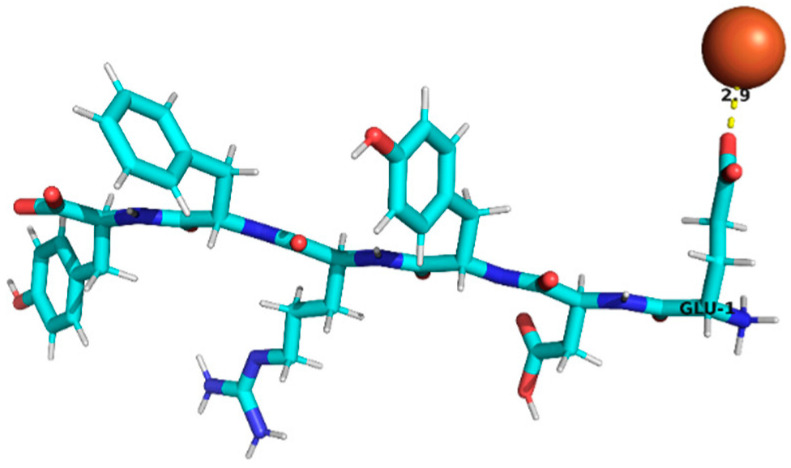
3	SNEPLYR	−0.55	Tyr 6 3.8 Å	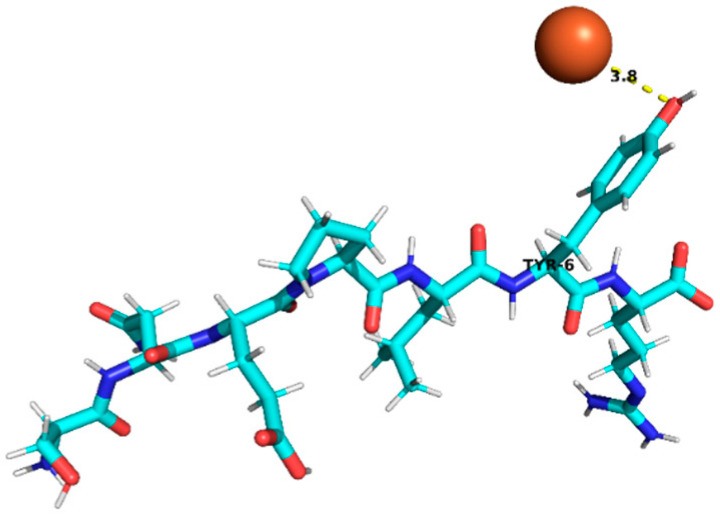
4	QQPLPR	−0.48	Leu 4 4.4 Å Pro 5 3.8 Å	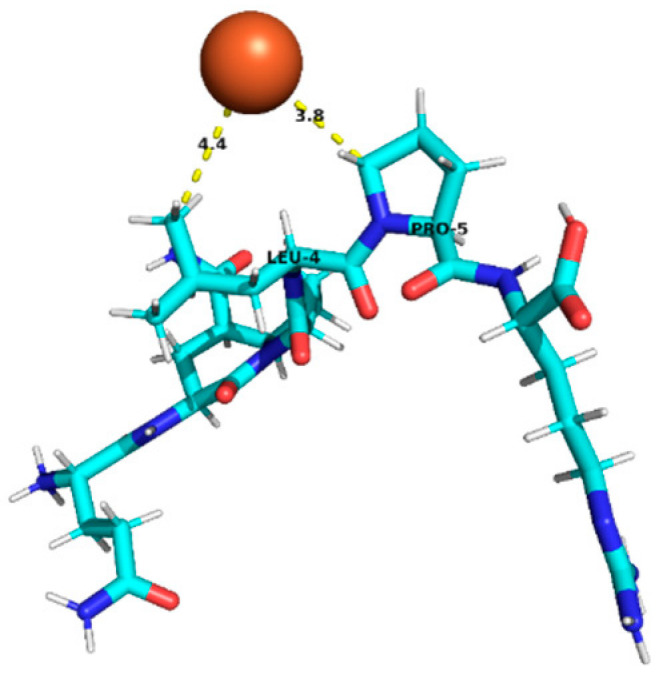
5	YDFLHF	−0.69	Phe 6 4.5 Å	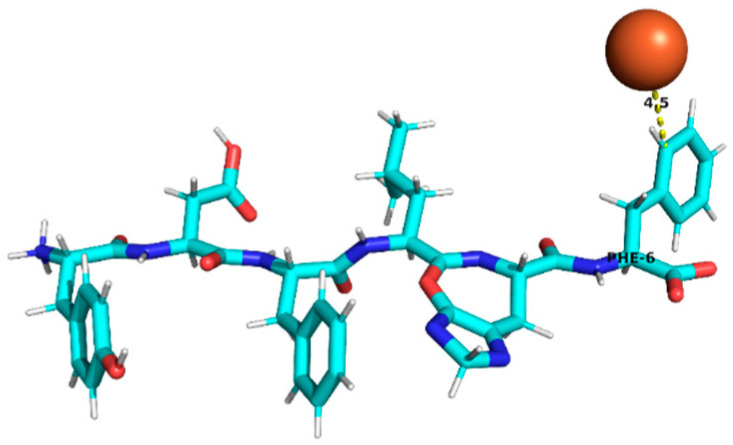
6	EPR	−0.46	Arg 3 5.9 Å	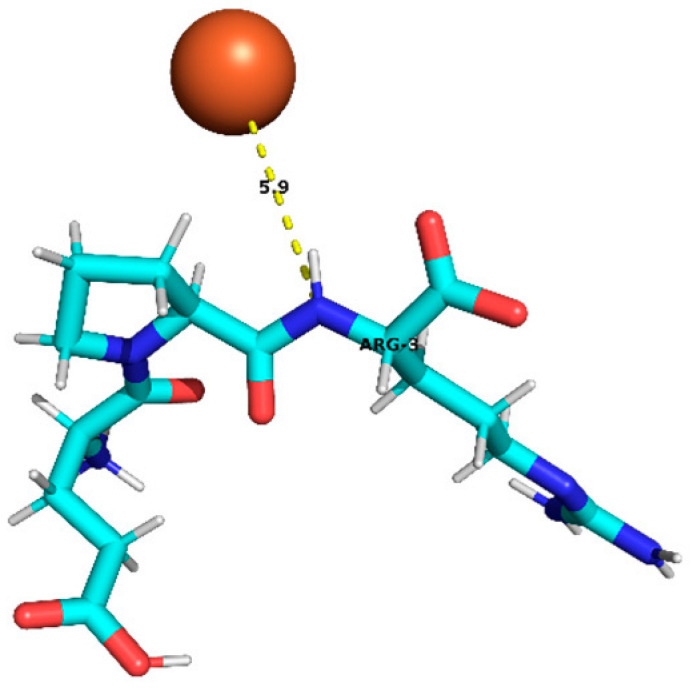
7	SPPTLRPR	−0.55	Leu 5 4.8 Å	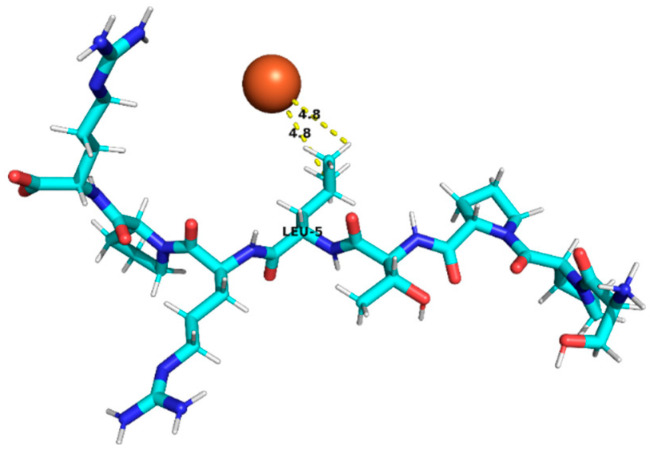
8	RYDRDGQLR	−0.42	Arg 4 7.0 Å	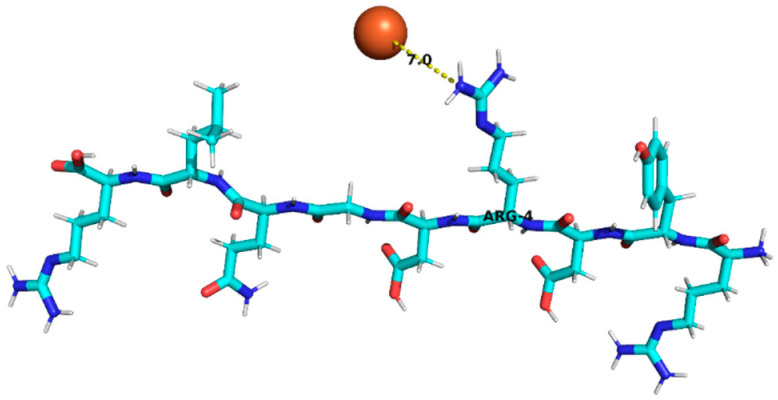
9	REPSLR	−0.51	Leu 5 8.1 Å	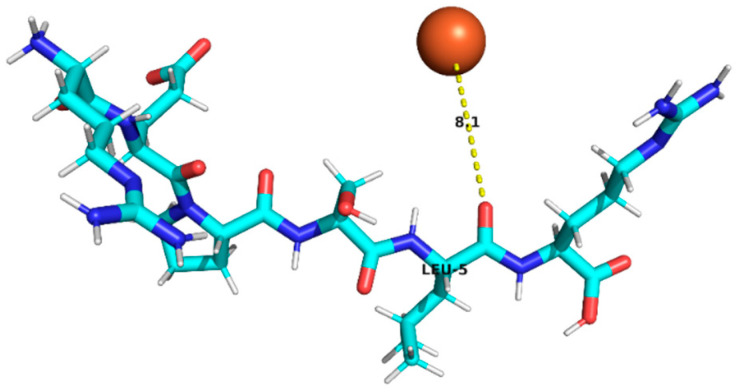

## Data Availability

The original contributions presented in the study are included in the article/Supplementary Materials. Further inquiries can be directed to the corresponding authors.

## Supplementary Materials

The following supporting information can be downloaded at: https://www.mdpi.com/article/10.3390/foods15081318/s1, Table S1: Peptides identified from the F2D fraction; Table S2: Peptides identified from the F3C fraction.
