# Supplementary material for: Discovery of Iron-Chelating Peptides from Lupinus mutabilis via Integrated Purification and In Silico Validation
Source: Foods. 2026 Apr 10;15(8):1318. doi: 10.3390/foods15081318 (PMC13115156; doi:10.3390/foods15081318)
Supplement: Supplementary file 1 [file foods-15-01318-s001.zip › foods-4207927-supplementary.pdf]

Table S1: Peptides identified from F2D

#	Scan #	m/z	z	Score	Peptide Mass	Error (ppm)	Length	De Novo Peptide	DB Sequence	protein
1	2519	421.249	2	98.4	840.4818	1.2	7	LVNPDKR	IVNPDKR	sp|F5B8V9|CONB1_LUPAN Conglutin beta 1 OS=Lupinus angustifolius OX=3871 GN=BETA1 PE=1 SV=1
2	1268	378.706	2	98.1	755.3966	1.2	6	YNLLPH		
3	980	353.709	2	97.6	705.4061	-3.6	6	YVLSPK		
4	1359	387.699	2	97.5	773.382	2	6	NDALWR		
5	927	393.215	1	97.3	392.206	4.3	3	NLF		
6	1001	381.203	2	97.1	760.3868	5.4	7	GLGFDPR		
7	2600	443.713	2	96.7	885.4093	2.8	7	NEGWQPR		
8	1438	379.206	2	96.7	756.3919	7	6	ADLWPR		
9	331	361.2	2	96.6	720.3806	5.5	6	SNKPLY	SNKPIY	sp|F5B8V9|CONB1_LUPAN Conglutin beta 1 OS=Lupinus angustifolius OX=3871 GN=BETA1 PE=1 SV=1
10	3272	378.226	2	96.5	754.4378	0.7	6	YLLPHL		
11	921	361.696	2	96.5	721.3759	2.6	6	SYPLSR		
12	1211	352.198	1	96.5	351.1907	-1	3	VPH		
13	1662	453.218	2	96.2	904.4192	2.6	7	FDGWQPR		
14	1000	385.255	1	96.1	384.2485	-0.9	3	LPR	LPR	tr|A0A1J7GVY1|A0A1J7GVY1_LUPAN Uncharacterized protein OS=Lupinus angustifolius OX=3871 GN=TanjilG_10508 PE=4 SV=1
15	2687	420.201	2	95.9	838.3861	2.4	6	DWYDLK		
16	1729	446.696	2	95.9	891.3763	2	6	EDYRFY		
17	460	359.22	2	95.9	716.4221	6	6	VKVPQF		
18	1777	410.194	2	95.7	818.3711	2.5	6	YQFPHQ		
19	2141	419.231	1	95.7	418.2216	5.1	3	LTW		
20	1178	419.697	2	95.6	837.377	2.2	6	NTWDRF		
21	1675	352.691	2	95.6	703.3653	3.3	6	LGDFPR		
22	2604	435.234	2	95.5	868.4515	1.8	7	LQNPNQR		
23	3327	401.182	2	95.5	800.3453	6.1	6	SDNFRY		
24	1809	387.2	1	95.5	386.1914	3.4	3	DPR		
25	3465	370.229	2	95.4	738.4428	0.4	6	FLLLPH		
26	2848	351.703	2	95.4	701.3861	7.4	5	VEWLR		
27	480	386.691	2	95.4	771.3664	2.1	6	EGWQPR		
28	2104	360.688	2	95.3	719.3602	0.6	6	FQSKPN	FQSKPN	sp|F5B8V9|CONB1_LUPAN Conglutin beta 1 OS=Lupinus angustifolius OX=3871 GN=BETA1 PE=1 SV=1
29	4227	353.236	2	95.3	704.4585	-2.1	6	LLLLPH		
30	3211	444.207	2	95.2	886.3974	3.2	7	YDGWLPH		
31	3593	353.716	2	95.2	705.4174	0	6	LNLLPH		
32	407	366.213	1	95.1	365.2063	-2.7	3	LPH		
33	1163	374.213	2	95	746.4075	4.2	7	TFPGGLR	FTPGGLR	tr|A0A4P1RQV7|A0A4P1RQV7_LUPAN Uncharacterized protein OS=Lupinus angustifolius OX=3871 GN=TanjilG_04563 PE=4 SV=1
34	255	359.228	1	95	358.2216	-1.5	3	LNL		
35	1278	415.203	2	94.8	828.3879	4.3	7	ADGWQPR		
36	3623	430.286	2	94.8	858.5579	-1.1	7	YLLLLPK		
37	622	435.26	1	94.8	434.2529	-0.5	4	VGLF		
38	3111	411.247	2	94.8	820.4807	-0.9	7	SVLFVTR		
39	3381	495.74	2	94.8	989.4607	4.3	8	YNGPWEPK		
40	1813	429.219	2	94.7	856.4192	5.5	7	VDGWQPR		
41	1485	350.207	1	94.3	349.2002	-0.8	3	LAF		
42	575	436.227	2	94.2	870.4388	1.8	7	FHPPLFN		
43	1564	354.69	2	94	707.3643	1.1	5	DWKLF		
44	2299	439.723	2	93.9	877.4294	2	7	SNEPLYR		
45	2680	364.212	2	93.6	726.4065	2.7	6	FTLLPH		
46	3388	422.212	2	93.6	842.4035	7.5	7	AEGWQPR		
47	1642	357.249	1	93.5	356.2424	-2.9	3	LPK		
48	1943	357.204	2	93.5	712.3908	4.6	6	FSLLPH		
49	342	379.689	2	93.5	757.3647	-1.9	6	YKDGLY		
50	479	386.691	2	93.4	771.3664	2.1	6	ADWQPR		
51	2827	352.721	2	93.2	703.4269	0.9	6	FSLLPK		
52	654	379.206	2	92.9	756.3959	2.9	6	FHPPLF		
53	2591	405.214	1	92.8	404.206	2.3	3	LSW		
54	3118	449.276	1	92.7	448.2686	-0.7	4	LGFL		
55	176	369.716	2	92.5	737.4184	-0.2	6	QQPLPR	QQPLPR	tr|A0A1J7GVY1|A0A1J7GVY1_LUPAN Uncharacterized protein OS=Lupinus angustifolius OX=3871 GN=TanjilG_10508 PE=4 SV=1
56	2959	365.745	2	92.4	729.4789	-4.3	6	FLLLPK		
57	2666	373.744	2	92.3	745.4738	-1.6	6	YLLLPK		
58	399	479.298	1	92	478.2904	0.5	4	LLPH		
59	1551	352.721	2	91.8	703.4269	0.5	6	SLLFPK		
60	1862	375.167	2	91.6	748.318	1.7	5	DWDKW		
61	3379	358.729	2	91.3	715.4381	8.3	6	FLRAPL		
62	1347	423.231	2	91.2	844.4443	4.7	8	SFPGVPNK		
63	2556	461.215	2	91.2	920.4141	2.1	7	YDGWQPR		
64	3529	424.225	2	91.1	846.4348	1	7	NPGQLYR		
65	916	436.227	2	91.1	870.4388	0.4	7	NFHPPLF		
66	3084	354.687	2	91	707.3602	-0.5	5	EYLRQ		
67	2072	396.72	2	90.8	791.429	-3.5	7	QGGYRLV		

68	1711	403.216	2	90.8	804.417	0.8	6	KLPDWF		
69	2598	410.208	1	90.8	409.2002	0.8	3	FPF		
70	1279	415.203	2	90.7	828.3879	4.3	7	ADGWQRP		
71	2373	356.23	2	90.6	710.4439	1.4	6	VLNRPL		
72	2028	422.21	2	90.5	842.4035	2.2	6	EQWQPR		
73	3594	353.716	2	90.4	705.4174	0	7	LGGLLPH		
74	2916	351.703	2	90.4	701.3861	8.3	5	LDWLR		
75	3358	420.716	2	90.3	839.4178	0.4	6	EPLYRY		
76	1886	443.713	2	90.2	885.4093	1.4	7	QDGWQPR		
77	826	399.26	1	90.2	398.2529	0.6	4	LAPV		
78	3471	390.737	2	90	779.4582	0.6	6	FYLLPK		
79	291	490.301	2	89.9	978.5862	0.9	9	VLSPPTLRP	VISPPTLRP	sp|F5B8V6|CONA1_LUPAN Conglutin alpha 1 OS=Lupinus angustifolius OX=3871 GN=CONALPHA PE=1 SV=1
80	1488	350.207	1	89.9	349.2002	-0.8	3	ALF		
81	3100	395.705	2	89.8	789.3922	3.7	6	FGWQPR		
82	1055	523.265	2	89.6	1044.5141	0.6	9	TFPGGWQPR		
83	3436	421.198	2	89.6	840.3806	1	6	YDFLHF		
84	3215	396.214	2	89.4	790.4086	5.2	6	NQRNFL	NQRNFL	sp|F5B8V9|CONB1_LUPAN Conglutin beta 1 OS=Lupinus angustifolius OX=3871 GN=BETA1 PE=1 SV=1
85	1277	415.203	2	89.4	828.3879	4.3	6	ENWQPR		
86	1883	352.708	2	89.3	703.4017	-0.5	6	FTRLPA		
87	2664	434.733	2	89.2	867.4491	2.8	7	SNKPLYF		
88	2168	359.203	1	89	358.1965	-3.1	3	SPR		
89	4202	438.732	2	89	875.4501	-0.7	7	YKNPQVQ		
90	2918	351.703	2	88.9	701.3861	8.3	5	WLDLR		
91	2425	451.249	2	88.9	900.4818	0.9	7	WDNVVLR		
92	444	366.214	1	88.8	365.2063	0.3	3	PLH		
93	445	366.214	1	88.6	365.2063	0.3	3	HPL		
94	1917	490.27	1	88.6	489.2587	7.3	4	FQVP		
95	582	413.277	1	88.4	412.2686	2.9	4	LAPL		
96	1102	517.277	1	88.4	516.2696	0.6	4	HLTF		
97	2509	393.54	3	88.3	1177.5952	1.9	9	RYDRDGQLR		
98	1643	425.709	2	88.1	849.4021	1.2	6	WEQKPY		
99	2541	429.715	2	87.8	857.4144	2.4	7	NTGWQPR		
100	3500	463.257	1	87.7	462.2478	3.1	4	LYAP		
101	1594	460.229	2	87.7	918.4348	8.4	7	FEGWQPR		
102	3839	430.706	2	87.6	859.4011	-4.7	7	DGVRMPW		
103	1555	364.724	2	87.5	727.4341	0.3	6	NQRVVL		
104	2319	380.218	1	87.3	379.2107	-0.4	3	LTF		
105	1090	359.71	2	87	717.4061	-0.4	6	TLPKPY		
106	3022	569.793	2	87	1137.5679	2.3	10	LNNPGGWQPR		
107	1167	374.213	2	86.9	746.4075	4.2	6	TFPRNL		
108	186	408.249	1	86.8	407.242	-0.1	3	LYL		
109	2892	366.168	1	86.8	365.1587	5.6	3	SPY		
110	3537	410.213	2	86.7	818.4109	-0.1	6	DWMVLR		
111	2219	364.707	2	86.7	727.3977	1.9	6	VNPDKR	VNPDKR	sp|F5B8V9|CONB1_LUPAN Conglutin beta 1 OS=Lupinus angustifolius OX=3871 GN=BETA1 PE=1 SV=1
112	766	433.209	1	86.7	432.2009	2.2	3	LDW		
113	2111	429.273	1	86.4	428.2635	4.1	4	LSLP		
114	1566	390.706	2	86.4	779.3966	0.1	6	TFPYPR		
115	2972	354.69	2	86.3	707.3643	1	6	YKPPGF		
116	1003	381.203	2	86.3	760.3868	5.4	7	GLGDFPR		
117	1950	363.7	2	86.2	725.3861	0.4	6	FVLHNP		
118	3318	413.236	2	86	824.4505	7.3	7	SQQPLPR		
119	2178	468.751	2	85.9	935.4865	1.3	7	DALWRLY		
120	557	416.732	2	85.8	831.4491	0.6	7	NTLPKPY		
121	3361	620.313	2	85.6	1238.6156	-3.3	10	TLNNPNWQPR		
122	1259	355.223	2	85.5	708.4283	3.4	7	AGPLRPV		
123	1902	353.172	2	85.3	704.3282	2.4	6	TFPGFH		
124	1719	352.199	2	85.3	702.3813	2.1	6	QFPVGR		
125	3630	413.728	2	85.3	825.4385	4.8	6	FDYLLR		
126	2939	478.813	2	85.2	955.6106	0.7	8	LLPLYLPK		

Table S2: Peptides idenfied from F3C

#	Scan #	m/z	z	Score	eptide Mas	Error (ppm	Length	De Novo Peptide	DB Sequence	Protein
1	1109	357.689	2	99.3	713.361	3.5	6	AGWQPR		
2	1342	352.691	2	99.1	703.365	1.8	6	LGFDPR		
3	1060	350.681	2	98.7	699.345	1.8	6	GGWQPR		
4	601	399.213	2	98.1	796.412	0.6	6	YNPLYK		
5	216	356.739	2	97.9	711.464	0.1	6	TLPLLR		
6	2290	453.744	2	97.6	905.472	2.7	7	FNDNLKR		
7	2559	360.2	1	97.2	359.192	2.9	3	GQR		
8	2123	394.197	2	96.8	786.377	3.7	7	SGGWQPR		
9	1679	364.707	2	96.7	727.398	1.9	6	VNPDKRQ	VNPDKR	sp|F5B8W0|CONB2_LUPAN Conglutin beta 2 OS=Lupinus angustifolius OX=3871 GN=BETA2 PE=1 SV=1
10	640	365.685	2	96.2	729.356	0.8	6	SGWQPR		
11	2273	408.215	2	96.1	814.409	9.6	6	TQWQPR		
12	1046	386.199	2	95.8	770.382	1.7	7	AGGWQPR		
13	1865	421.249	2	95.8	840.482	1.2	7	LVNPDKR	IVNPDKR	sp|F5B8W0|CONB2_LUPAN Conglutin beta 2 OS=Lupinus angustifolius OX=3871 GN=BETA2 PE=1 SV=1
14	1235	395.704	2	94.8	789.392	0.4	6	FGWQPR		
15	504	401.215	1	94.5	400.207	1.7	3	EPR		
16	615	357.688	2	94.3	713.361	0.3	5	QWQPR		
17	1714	439.723	2	94	877.429	2	7	SNEPLYR		
18	651	378.711	2	94	755.408	-0.6	6	LGWQPR		
19	1021	381.203	2	93.9	760.387	5.1	7	GLGFDPR		
20	284	435.236	1	93.7	434.231	-6.5	3	M(O)LR		
21	706	372.693	2	93.3	743.372	0.4	6	TGWQPR		
22	737	385.255	1	93.1	384.249	-0.9	3	LPR		
23	1625	454.712	2	93.1	907.408	1.9	8	GSSFYYGK		
24	471	386.199	2	93.1	770.382	2.5	6	GQWQPR		
25	1365	403.702	2	92.7	805.387	2.5	6	YGWQPR		
26	1878	429.715	2	92.7	857.414	2.4	7	NTGWQPR		
27	2230	391.72	2	92.5	781.424	1.9	6	LHPYPR		
28	2186	439.22	2	92.2	876.424	1.8	7	FSGWQPR		
29	2011	415.217	2	91.9	828.424	-5.1	7	VSGWQPR		
30	129	462.275	2	91	922.535	0.5	8	SPPTLRPR	SPPTLRPR	sp|F5B8V6|CONA1_LUPAN Conglutin alpha 1 OS=Lupinus angustifolius OX=3871 GN=CONALPHA PE=1 SV=1
31	1877	429.715	2	91	857.414	2.4	7	QSGWQPR		
32	2481	374.866	3	90.7	1121.57	2.1	9	KDVHQPGWR		
33	787	523.265	2	90.6	1044.51	0.6	9	TFPGGWQPR		
34	2677	361.192	2	90.4	720.367	3.1	5	RFDQR	RFDQR	sp|F5B8W0|CONB2_LUPAN Conglutin beta 2 OS=Lupinus angustifolius OX=3871 GN=BETA2 PE=1 SV=1
35	2607	353.686	2	90.2	705.356	1.8	5	RDYPR		
36	1164	407.222	2	90.1	812.429	-0.6	7	LGGWQPR		
37	760	350.68	2	90	699.345	0.5	5	NWQPR		
38	1415	358.196	2	89.7	714.381	-4.9	6	SWLGPR		
39	257	413.732	2	89.7	825.45	0.5	7	SWLPAPR		
40	898	407.205	1	89.4	406.2	-5.6	3	MTR		
41	2591	410.716	2	89.1	819.413	6.2	7	ADLYNPK		
42	984	415.203	2	88.9	828.388	4.3	7	DAGWQPR		
43	25	379.236	3	88.1	1134.69	-0.5	10	VLSPPTLRPR	VISPPTLRPR	sp|F5B8V6|CONA1_LUPAN Conglutin alpha 1 OS=Lupinus angustifolius OX=3871 GN=CONALPHA PE=1 SV=1
44	1981	423.217	2	87.6	844.419	-0.8	7	TTGWQPR		
45	2782	409.731	2	87.2	817.445	3.6	7	SSPKFPR		
46	2624	355.75	2	86.9	709.485	1.8	6	LPLVLR		
47	1786	426.201	2	86.2	850.386	1.1	7	SSFYYGK		
48	1856	393.54	3	85.7	1177.6	1.9	9	RYDRDGQLR		
49	1962	379.22	2	85.4	756.424	0.3	6	REPSLR		
50	2217	478.285	2	85. 3	954.547	7.8	7	RDKRQPR		
